# Validation of the Japanese Version of the Multidimensional Measure of Family Supportive Supervisor Behaviors (FSSB-J)

**DOI:** 10.3389/fpsyg.2019.02628

**Published:** 2019-11-22

**Authors:** Hisashi Eguchi, Yuko Kachi, Hayami K. Koga, Mariko Sakka, Masahito Tokita, Akihito Shimazu

**Affiliations:** ^1^Department of Public Health, Kitasato University School of Medicine, Sagamihara, Japan; ^2^Department of Social and Behavioral Sciences, Harvard T.H. Chan School of Public Health, Boston, MA, United States; ^3^Division of Health Sciences and Nursing, Department of Gerontological Home-Care and Long-Term Care Nursing, Graduate School of Medicine, The University of Tokyo, Tokyo, Japan; ^4^Keio Research Institute at SFC, Keio University, Fujisawa, Japan; ^5^Faculty of Policy Management, Keio University, Fujisawa, Japan; ^6^Asia Pacific Centre for Work Health and Safety, University of South Australia, Adelaide, SA, Australia

**Keywords:** workplace, psychosocial factor, Japan, supervisor behavior, work–life balance, scale validation

## Abstract

**Objective:**

The aim of the current study was to validate the Japanese version of the family supportive supervisor behaviors (FSSB-J) measure. FSSB is conceptualized as a multidimensional, superordinate construct constituted of four dimensions: emotional support, instrumental support, role modeling behaviors, and creative work–family management.

**Methods:**

The Japanese translated and back-translated FSSB-J questionnaire was administered to 1,670 men and women aged 20–59 years who were registered with a Japanese online survey company in November 2017. Confirmatory factor analyses were performed to evaluate the factorial validity of the FSSB-J. Cross-time measurement invariance was tested using multi-group confirmatory factor analyses. Construct validity was assessed with the potential consequences of FSSB (e.g., work–family spillover, work engagement, intention to leave, job satisfaction, and psychological distress) and convergent validity was assessed using similar concepts (e.g., organizational justice and social support). Internal consistency and test–retest reliability were examined to evaluate the reliability of the four dimensions of the FSSB.

**Results:**

A series of confirmatory factor analyses using the multiple-group method revealed that the four-factor model fitted the data best. The latent factor structure demonstrated configural, metric, and scalar invariance across time. Construct and convergent validity were generally in line with expectations. Cronbach’s α coefficient and test–retest reliability were sufficient for each of the four dimensions of the FSSB.

**Conclusion:**

This study suggests that FSSB-J is an adequate measure of FSSB in the Japanese context.

## Introduction

In most contemporary developed countries, many households are financially supported by dual-earner couples, with approximately 60% of couples supporting families together (Organisation for Economic Co-operation and Development, 2011). With an increase in the proportion of dual-earner families, work and non-work demands are now more likely to conflict (Shimazu et al., 2010; Cabinet Office, 2018; French et al., 2018). For dual-earner families, it has become important to maintain an appropriate balance between work and family roles. Japan is unique in terms of its strong corporate culture, in which employees are judged by their willingness to spend extended hours face-to-face with co-workers, being required to attend endless successions of meetings, and expected to prioritize work over private life. These societal features make the implementation of work–family balance policies particularly challenging in Japan (Cabinet Office, 2018).

Work–family balance is particularly critical in Japan, where fertility rates have plummeted over the last several years to the lowest level of any country, contributing to rapid population decline (Japan Ministry of Health Labour and Welfare, 2017). Since previous studies have found that the promotion of work and family–life balance is associated with improved fertility rates, this may be an important point of intervention (Yamaguchi and Youm, 2012). As an attempt to combat this decline in birthrate, and, subsequently, the population, the Japanese government has recently promoted a reform of working styles known as “Work-style Reform.” This reform includes the introduction of legal caps on overtime hours, rules to establish the “equal work, equal pay” principle by improving working conditions for people with irregular employment status, and a new system that allows corporate employees to be paid based on their performance rather than on the number of hours spent in the workplace. The promotion of work and family–life balance is an important part of this proposed work-style reform (Japan Ministry of Health Labour and Welfare, 2017).

Work–family research has identified social support from supervisors as an important resource that can reduce the negative effects of work and family stressors (O’Driscoll et al., 2003). Moreover, the Japanese government has started the “iku boss” (i.e., educating the boss) system to re-educate managers about the need to support the work–family balance of their subordinates (Japan Ministry of Health Labour and Welfare, 2017). To help promote this change, specific measures for assessing supervisors’ behaviors in encouraging work–family balance among their subordinates are needed in Japan.

Family supportive supervisor behaviors (FSSB) are defined as behaviors exhibited by supervisors that are supportive of the family roles of employees (Hammer et al., 2009). FSSB was originally conceptualized as a multidimensional superordinate construct which is assessed with 14 items that construct the four dimensions; emotional support, instrumental support, role modeling behaviors, and creative work–family management. This measure has been shown to have sufficient construct, criterion-related, and incremental validity. It has been significantly associated with work–family conflict, work–family positive spillover, job satisfaction, and turnover intentions over and above measures of general supervisor support (Hammer et al., 2009). Furthermore, FSSB has been linked to a number of employee outcomes, including lower levels of turnover intentions and higher levels of job satisfaction (Hammer et al., 2011; Crain et al., 2014; Odle-Dusseau et al., 2016; Yragui et al., 2017). To investigate whether the application of FSSB in Japan is appropriate, the validation of a Japanese version of FSSB is a necessary first step.

Higher levels of supervisor support can benefit employees, and is related to higher levels of work–family positive spillover (Thompson et al., 1999). In addition, studies have found that supervisor support enhances employee job satisfaction (Steinhardt et al., 2003; Odle-Dusseau et al., 2016), and lessens turnover intentions (Nichols et al., 2016; Fukui et al., 2019) and psychological distress (Winnubst et al., 1982; Kawakami et al., 2005). Therefore, it was hypothesized that FSSB is positively associated with work–family positive spillover and job satisfaction, and negatively associated with turnover intentions, which would confirm the construct validity of the scale.

### The Current Study

This study aimed to validate the Japanese version of the FSSB (FSSB-J) in a sample of Japanese employees. Specifically, the factorial and construct validity of this measure were examined, as well as its reliability (i.e., internal consistency and test–retest reliability). The development of the Japanese version of the FSSB scale was based on the COnsensus-based Standards for the selection of health Measurement INstruments (COSMIN) (Mokkink et al., 2018).

## Materials and Methods

### Translation of the FSSB

Professor Leslie Hammer, the author of the original version of FSSB, approved the development of the FSSB-J. First, the English version of the FSSB was translated into Japanese by two of the authors of this study (HE and YK), then back-translated to English by one author of this study (HK) who was blinded to the original items and is proficient in both English and Japanese. The back-translated version was confirmed by Professor Leslie Hammer. The original English version was compared with the back-translated version of the FSSB and created a preliminary Japanese version after terminology corrections.

### Study Design and Population

A series of online surveys was administered to men and women who were registered with a Japanese online survey company in December 2017. Of all workers who were registered with this online survey company, 1,670 married workers who were 20–59 years of age were selected and invited by e-mail to complete a web-based questionnaire in the order of arrival. The online survey company had access to more than 2,000,000 potential participants who showed interest in participating in surveys that provided fiscal incentives. The fiscal incentive for this survey was low, valued at several U.S. dollars. For financial reasons, recruitment stopped when the number of participants reached 1,670. The survey was repeated with the same respondents 1 month later because an interval between surveys of approximately 1 month is typically considered appropriate for the evaluation of instruments (Streiner et al., 2015). Respondents in the second survey were recruited from participants who completed the first survey on a first-come first-served basis. The recruitment procedure in the second survey was the same as the first survey. Recruitment ceased when the number of participants reached 1,000. There were no statistically significant differences in FSSB scores across the participant demographic characteristics between the first and second surveys (Table 1).

**TABLE 1:** Participant demographics and means and deviations of total FSSB score.

	**Baseline survey (*n* = 1,378)**				**Follow-up survey (*n* = 842)**			
		
	***n***	**(%)**	**Mean**	**SD**	***n***	**(%)**	**Mean**	**SD**
**Sex**								
Men	965	(70.0)	41.2	(12.9)	597	(70.9)	39.3	(11.6)
Women	413	(30.0)	42.5	(14.0)	245	(29.1)	41.5	(12.5)
**Age (years)**								
20–29	27	(2.0)	43.1	(2.0)	14	(1.7)	42.6	(7.4)
30–39	279	(20.3)	44.6	(20.1)	192	(22.8)	42.4	(11.5)
40–49	532	(38.6)	41.2	(38.6)	319	(37.9)	39.8	(11.8)
50–59	540	(39.2)	40.4	(39.2)	317	(37.6)	38.5	(12.1)
**Educational status**								
Junior high school	7	(0.51)	45.7	(14.1)	2	(0.2)	46.0	(2.8)
High school	410	(29.8)	39.6	(13.3)	251	(29.8)	38.6	(11.9)
College	164	(11.9)	43.2	(13.2)	90	(10.7)	40.8	(12.7)
University	672	(48.8)	42.3	(13.2)	421	(50.0)	40.4	(11.8)
Graduate school	124	(9.0)	42.2	(12.8)	77	(9.1)	41.0	(11.3)
Others	1	(0.1)	44.0	(0.0)	1	(0.1)	37.0	(0)
**Having children**								
Yes	1080	(78.4)	41.4	(13.3)	657	(78.0)	39.7	(11.7)
No	298	(21.6)	42.5	(13.2)	185	(22.0)	40.8	(12.7)
**Occupation**								
Professional and technical job	330	(24.0)	40.7	(13.1)	215	(25.5)	39.5	(11.5)
Managerial job	220	(16.0)	43.0	(13.3)	131	(15.6)	40.1	(11.7)
Clerical job	342	(24.8)	43.1	(13.6)	217	(25.8)	41.5	(11.7)
Sales job	159	(11.5)	41.2	(13.1)	93	(11.0)	39.8	(12.6)
Service job	92	(6.7)	41.7	(13.1)	47	(5.6)	40.8	(12.4)
Manufacturing job	112	(8.1)	38.0	(13.1)	68	(8.1)	36.4	(13.2)
Security job	29	(2.1)	45.9	(12.2)	17	(2.0)	42.7	(9.3)
Agriculture, forestry, and fishery job	4	(0.3)	43.0	(13.4)	1	(0.1)	62.0	(0.0)
Transportation and communication job	42	(3.1)	39.3	(12.2)	28	(3.3)	35.2	(11.9)
Others	48	(3.5)	40.0	(12.8)	25	(3.0)	42.0	(9.4)
**Employment status**								
Regular	1081	(78.5)	41.5	(13.2)	678	(80.5)	39.5	(11.8)
Non-regular	297	(21.6)	42.2	(13.4)	164	(19.5)	41.7	(12.3)
**Weekly working hours**								
−19	223	(16.2)	41.5	(14.6)	126	(15.0)	39.0	(12.4)
20–39	233	(16.9)	42.5	(13.1)	136	(16.2)	42.4	(12.1)
40–59	762	(55.3)	41.8	(12.9)	473	(56.2)	40.1	(11.6)
60–79	136	(9.9)	39.3	(13.2)	89	(10.6)	37.8	(11.7)
80–	24	(1.7)	40.2	(14.6)	18	(2.1)	37.2	(12.9)

A total of 249 participants were excluded from the first survey and 135 were excluded from the second survey because they reported being self-employed or freelance workers. A further 43 participants who had at least one missing response on the questionnaire in the first survey were excluded, resulting in a final sample of 1,378 respondents for the first survey and 842 respondents for the second survey. COSMIN requires the researcher to evaluate the percentage of missing items because a high number of missing items can introduce bias in the study results. In the online survey, it was possible to restrict the participants from answering all questions. To evaluate bias, the restriction was removed only for FSSB items in the first survey. Since the online survey required participants to answer all questions except for the FSSB items in the first survey, no participants had missing data on the FSSB items in the second survey, or for the non-FSSB items in both surveys.

### Measures

#### Family Supportive Supervisor Behavior

Family Supportive Supervisor Behavior was assessed with a preliminary Japanese version of the FSSB measure. The items of the FSSB measure are grouped into four subscales that reflect the underlying dimensions of FSSB: emotional support (five items), role modeling behaviors (three items), instrumental support (four items), and creative work–family management (six items). All items are scored on a five-point Likert scale ranging from 1 (“strongly disagree”) to 5 (“strongly agree”). The total score of the subscales was used in the analyses with higher scores representing higher levels of the construct.

#### Procedural and Interactional Justice

Two aspects of organizational justice (i.e., procedural and interactional) were assessed using the Japanese version of the Organizational Justice Questionnaire (OJQ). This scale was developed by Moorman (1991) and modified by Elovainio et al. (2002). The scale comprises of a seven-item scale that measures procedural justice, and a six-item scale that measures interactional justice, both of which are rated on a five-point Likert scale ranging from 1 (“strongly disagree”) to 5 (“strongly agree”). The total score for each OJQ subscale was calculated by averaging item scores (score range 1–5). The subscale scores are summed to calculate the total score, where higher scores indicate higher levels of organizational justice. This Japanese version has been shown to have sufficient reliability and validity (Inoue et al., 2009). In the present study sample, Cronbach’s α coefficients for the procedural and interactional justice scales were 0.88 and 0.94, respectively.

#### Source-Specific Workplace Social Support

To assess source-specific workplace social support, the New Brief Job Stress Questionnaire (New BJSQ) (Shimomitsu, 2000) was used. The New BJSQ includes a three-item supervisor and coworker support scale (response range: 3–12), with items such as “How freely can you talk with the supervisor/coworker?,” “How reliable is the supervisor/coworker when you are troubled?,” and “How well will the supervisor/coworker listen to you when you ask for advice on personal matters?” Participants responded to these statements on a four-point Likert scale ranging from 1 (“Extremely”) to 4 (“not at all”). The item scores are summed to calculate the total score. The higher scores indicate the greater levels of supervisor and co-worker support. The Cronbach’s α coefficient was 0.91 for supervisor support and 0.80 for co-worker support.

#### Work–Family Positive Spillover

Work–family positive spillover effects [work-to-family positive spillover (WFPS) and family-to-work positive spillover (FWPS)] were measured with 22 items using the Survey Work–Home Interaction–NijmeGen (SWING), which was developed in the Netherlands (Geurts et al., 2005). WFPS was measured with four items (e.g., “You manage your time at home more efficiently as a result of the way you do your job”) (response range: 0–12). FWPS was measured with four items (e.g., “After spending a pleasant weekend with your spouse/family/friends, you have more fun in your job”) (response range: 0–12). Items are scored on a four-point Likert scale, ranging from 0 (“never”) to 3 (“always”). The item scores are summed to calculate the total score, with higher scores indicating greater levels of WFPS and FWPS. The reliability and validity of the Japanese version of the scale has previously been shown to be sufficient (Shimada et al., 2019). The Cronbach’s α coefficients in the sample were 0.72 for WFPS and 0.81 for FWPS.

#### Work Engagement

Work engagement was assessed using the short form of the Utrecht Work Engagement Scale (UWES) (Schaufeli et al., 2006). The UWES includes three subscales that reflect the underlying dimensions of engagement: vigor, dedication, and absorption (three items for each dimension). All items were scored on a 7-point Likert scale ranging from 0 (“never”) to 6 (“always”) (response range: 0–54). The item scores are summed to calculate the total score. Higher scores indicate greater levels of work engagement. A previous validation study of the Japanese version of the UWES recommended that work engagement should be treated as a unitary construct due to the high correlations among the three components (Shimazu et al., 2008). In the sample, Cronbach’s α coefficient was 0.93 for the UWES.

#### Intention to Leave

Intention to leave was measured with a three-item scale used by Geurts et al. (1998). Respondents used a five-point Likert scale ranging from 1 (“I agree completely”) to 5 (“I disagree completely”) to rate the extent to which they have the intention to leave their current employment in the following month (response range: 3–15). The item scores are summed to calculate the total score. Higher scores indicate greater levels of intention to leave. The internal consistency reliability of this measure was found to be good in the sample (α = 0.85).

#### Job Satisfaction

Job satisfaction was measured using the subscales of the New BJSQ (Inoue et al., 2014). Job satisfaction, which was a single item measure, was classified into four categories; 1 = dissatisfied, 2 = somewhat dissatisfied, 3 = relatively satisfied, or 4 = satisfied.

#### Psychological Distress

The K6 scale was developed by Kessler et al. (2002). The scale consists of six items that measure the extent of psychological distress using a five-point response option 0 (“none of the time”) to 4 (“all of the time”). The Japanese version of the K6 scale has been shown to have sufficient reliability and validity (Furukawa et al., 2008). The item scores are summed to calculate the total score. Higher scores indicate greater levels of psychological distress. In the current study, the Cronbach’s α coefficient for the K6 scale was 0.88.

#### Sociodemographic Factors

Sociodemographic factors included sex, age range (i.e., 20–29, 30–39, 40–49, and 50–59 years), educational attainment (junior high school, high school, junior college/technical school, university, graduate school, and others), having children (yes, no), occupation (professional and technical job, managerial job, clerical job, sales job, service job, manufacturing job, security job, agriculture, forestry or fishery job, transportation and communication job, others), employment contract (regular or part-time), and weekly working hours (≤19, 20–39, 40–59, 60–79, ≥80).

#### Hypothesis Testing

COnsensus-based Standards for the selection of health Measurement INstruments suggests that hypothesis testing should be used when a gold standard of a construct is not available (Mokkink et al., 2018) and FSSB is theoretically related to other psychosocial factors in the workplace (Hammer et al., 2009). Based on previous studies (Hammer et al., 2009), it was hypothesized that FSSB is negatively correlated with intention to leave and psychological distress, and positively correlated with procedural justice, interactional justice, supervisor support, coworker support, work–family positive spillover, family–work positive spillover, work engagement, and job satisfaction.

### Statistical Analysis

Classical test theory was used to measure FSSB. Mean values and standard deviations were calculated for each item of the FSSB scale. Item-total Spearman’s correlations were examined. CFA using maximum-likelihood estimation was conducted with AMOS (Chicago, IL, United States). The hypothesized four-factor model (Model 2) was used with a one-factor model (Model 1), whereby all items were loaded on one general FSSB factor. Moreover, to consider gender differences in the model, model testing was carried out in male and female samples simultaneously using the multiple-group method (Model 3 for the one-factor model and Model 4 for the four-factor model). Model fit was assessed using a combination of fit indices including the goodness of fit index (GFI), parsimony goodness of fit index (PGFI), non-normed fit index (NNFI), the comparative fit index (CFI), parsimony normed fit index (PNFI), and the root mean square error of approximation (RMSEA). The acceptability of model fit was judged by the following criteria; GFI, PGFI, NNFI, CFI, and PNFI of >0.90 and RMSEA of <0.08 (Hooper et al., 2008).

The survey was repeated after 1 month using the same FSSB scale on 1,000 of the 1,670 respondents who completed the first survey to assess the replicability of the FSSB with the test–retest method. To evaluate the cross-time measurement invariance, configural, metric, and scalar invariance were evaluated in three steps. First, configural invariance was tested using a two-level (i.e., first and second survey) CFA in which all parameters were estimated freely, except for the highest loading items for each factor, which were set to 1.0, while the factor means were set to 0. Second, metric invariance was tested by constraining all factor loadings to be equal for the two levels. Factor variances were set to 1.0 to identify the model. Third, scalar invariance was tested by constraining all subscale and item intercepts to be equal for the two levels. Model fit was assessed using a combination of fit indices including the Tucker–Lewis index (TLI), the CFI, and the RMSEA. The overall scale reliability was quantified by both an intraclass correlation coefficient (ICC) (2,1) based on a single measurement two-way random effects model of absolute agreement and the standard error of measurement (SEM). ICC (2,1) was red in compliance with published recommendations; an ICC (2,1) of 0.90 or higher was considered excellent, 0.75 or higher was considered good, 0.50 or higher was considered moderate, and lower than 0.50 was considered poor (Shrout and Fleiss, 1979). At least 301 participants were deemed to be necessary to detect an ICC (2,1) ≥ 0.50 (error α = 0.05 and β = 0.20) between the first and second survey (Walter et al., 1998). Previous studies indicate that this is a modest sample size (Terwee et al., 2012). Larger SEM represents lower test reliability and less precision in the measures and scores obtained (de Vet et al., 2006). Cronbach’s α coefficient was calculated to evaluate internal consistency and Pearson’s correlation coefficients were calculated to evaluate construct validity and convergent validity. All analyses were conducted in Stata 15 (College Station, TX, United States) and AMOS Version 25 for Windows (Chicago, IL, United States).

### Ethics Statement

This study was approved by the Ethics Committee of the Graduate School of Medicine and Faculty of Medicine at The University of Tokyo [11666-(1)].

## Results

The majority of the sample at baseline was male (70.0%), had more than high school education (69.7%), had children (78.4%), had regular employment status (78.5%), and worked >40 h per week (66.9%) (Table 1).

### Validity

A confirmatory factor analysis was conducted. As shown in Table 2, the hypothesized four-factor model (Model 2: each item loads on a hypothesized factor) showed a significantly better fit to the data than the one-factor model (Model 1: all items measuring the four constructs load on one general FSSB factor). The fit indices of the four-factor model (Model 2) were GFI = 0.923, PGFI = 0.624, NNFI = 0.961, CFI = 0.964, PNFI = 0.750, and RMSEA = 0.083. The fit indices of the four-factor model using a multiple-group model (Model 4) were GFI = 0.918, PGFI = 0.621, NNFI = 0.957, CFI = 0.964, PNFI = 0.747, and RMSEA = 0.059. A formal χ^2^ difference test revealed that the difference between the two models was not significant [Δχ^2^(71) = 73.265, *p* > 0.05]. These results suggest that factor loadings were indeed invariant between males and females.

**TABLE 2:** Results of confirmatory factor analyses: comparison of goodness-of-fit indices among one-factor models and four-factor models.

**Model**	**GFI**	**PGFI**	**NNFI**	**CFI**	**PNFI**	**RMSEA**	**χ^2^**	**df**	***p***
Model 1 (one-factor model)	0.659	0.483	0.816	0.819	0.690	0.179	3511.19	77	<0.01
Model 2 (four-factor model)	0.923	0.624	0.961	0.964	0.750	0.083	746.987	71	<0.01
Model 3 (one-factor model) (multiple-group method)	0.658	0.483	0.813	0.820	0.688	0.126	3567.96	154	<0.01
Model 4 (four-factor model) (multiple-group method)	0.918	0.621	0.957	0.964	0.747	0.059	820.252	142	<0.01

*GFI, goodness of fit index; AGFI, adjusted goodness of fit index; NNFI, non-normed fit index; CFI, comparative fit index; PNFI, parsimony normed fit index; RMSEA, root mean square error of approximation.*

Construct validity was tested by exploring the associations between the FSSB score and theoretically related constructs, including procedural justice, interactional justice, supervisor support, coworker support, WFPS, FWPS, work engagement, intention to leave, and psychological distress. Table 3 shows that FSSB scores were significantly positively correlated with procedural justice, interactional justice, supervisor support, coworker support, WFPS, FWPS, and work engagement, and negatively correlated with intention to leave and psychological distress (all *p* < 0.01).

**TABLE 3:** Association between family supportive supervisor behavior score and other constructs at baseline.

**Other constructs**	**Correlation coefficients^a^**				
	
	**Overall scale**	**Emotional support**	**Instrumental support**	**Role modeling**	**Creative work-family**
Procedural justice	**0.52**	**0.44**	**0.46**	**0.46**	**0.51**
Interactional justice	**0.73**	**0.66**	**0.68**	**0.61**	**0.69**
Supervisor support	**0.60**	**0.59**	**0.56**	**0.47**	**0.52**
Coworker support	**0.30**	**0.29**	**0.30**	**0.21**	**0.25**
WFPS	**0.21**	**0.18**	**0.20**	**0.19**	**0.18**
FWPS	**0.20**	**0.18**	**0.18**	**0.18**	**0.17**
Work engagement	**0.35**	**0.31**	**0.31**	**0.30**	**0.33**
Intention to leave	**−0.35**	**−0.30**	**−0.33**	**−0.30**	**−0.32**
Job satisfaction	**0.39**	**0.35**	**0.36**	**0.32**	**0.36**
Psychological distress	**−0.10**	−0.09	−0.14	−0.05	−0.07

*^*a*^Pearson’s correlations. WFPS, work-family positive spillover; FWPS, family-work positive spillover. Bold values denote statistical significance at the *p* < 0.05 level.*

### Reliability

Table 4 shows the means, standard deviations, item-total correlations, and Cronbach’s α coefficients of the scale when each item was removed. The mean score for each item was between 2.5 and 3.1. Correlations between items varied from 0.52 to 0.85 (*p* < 0.01). The item-total correlations ranged from 0.75 to 0.87 (*p* < 0.01). The correlations between the FSSB scores of the first and second surveys indicated high test–retest reliability (*r* = 0.72, *p* < 0.01). Table 5 shows the results of configural, metric, scalar invariance across time. In line with criteria defined by Bentler and colleagues (CFI > 0.900, RMSEA < 0.080) (Bentler, 1990; Hu and Bentler, 1999), the latent factor structure appeared to demonstrate configural, metric, and scalar invariance across time. The ICC (2,1) and SEM for FSSB were 0.70 [95% confidence interval (CI): 0.66–0.73] and 2.58 (95% CI: 2.52–2.64). The mean time variation for the FSSB was 0.03 ± 0.005. The internal consistency of the whole scale with 14 items was excellent, with a Cronbach’s α coefficient of 0.93. Omission of any of the items did not substantially alter the internal reliability of the scale. Cronbach’s alpha values were 0.94 for emotional factors, 0.90 for instrument support, 0.92 for role model, and 0.91 for creative work–family management.

**TABLE 4:** Means, standard deviations, item-total correlations, and Cronbach’s α if item deleted.

		**Means**	**SD**	**Item-total**	**Cronbach’s α**
				**correlation**	**if item deleted**
	Emotional support				
Item 1	My supervisor is willing to listen to my problems in juggling work and non-work life.	2.88	(1.16)	0.81	0.96
Item 2	My supervisor takes the time to learn about my personal needs.	2.86	(1.12)	0.82	0.96
Item 3	My supervisor makes me feel comfortable talking to him or her about my conflicts between work and non-work.	2.67	(1.06)	0.87	0.96
Item 4	My supervisor and I can talk effectively to solve conflicts between work and non-work issues.	2.73	(1.08)	0.85	0.96
	Instrument support				
Item 5	I can depend on my supervisor to help me with scheduling conflicts if I need it.	3.03	(1.15)	0.79	0.96
Item 6	I can rely on my supervisor to make sure my work responsibilities are handled when I have unanticipated non-work demands.	3.12	(1.13)	0.76	0.96
Item 7	My supervisor works effectively with workers to creatively solve conflicts between work and non-work.	2.90	(1.05)	0.82	0.96
	Role model				
Item 8	My supervisor is a good role model for work and non-work balance.	2.63	(1.05)	0.75	0.96
Item 9	My supervisor demonstrates effective behaviors in how to juggle work and non-work balance.	2.63	(1.01)	0.78	0.96
Item 10	My supervisor demonstrates how a person can jointly be successful on and off the job.	2.53	(0.98)	0.75	0.96
	Creative work–family management				
Item 11	My supervisor thinks about how the work in my department can be organized to jointly benefit employees and the company.	2.83	(1.09)	0.76	0.96
Item 12	My supervisor asks for suggestions to make it easier for employees to balance work and non-work demands.	2.56	(1.04)	0.80	0.96
Item 13	My supervisor is creative in reallocating job duties to help my department work better as a team.	2.81	(1.10)	0.78	0.96
Item 14	My supervisor is able to manage the department as a whole team to enable everyone’s needs to be met.	2.75	(1.08)	0.80	0.96

*SD, standard deviation; item-total correlations: all Spearman correlations; *p* < 0.01 in all cases.*

**TABLE 5:** Evaluation of measurem ent invariance: comparison of goodness-of-fit indices among configural invariance, metric invariance, and scalar invariance.

**Model**	**TLI**	**CFI**	**RMSEA**	**χ^2^**	**df**
Configural invariance					
Model 1	0.949	0.965	0.051	1168.29	142
Metric invariance					
Model 2	0.952	0.965	0.049	1179.37	152
Δ in fit from Model 1	0.003	0.000	–0.002	11.08	10
Scalar invariance					
Model 3	0.956	0.965	0.047	1202.14	166
Δ in fit from Model 2	0.004	0.000	–0.002	22.77	14

*TLI, Tucker–Lewis index; CFI, comparative fit index; RMSEA, root mean square error of approximation.*

## Discussion

The current results revealed that the Japanese version of the FSSB has good reliability, construct validity, and convergent validity, as well as adequate structural validity. In accordance with the COSMIN model, acceptable levels of internal consistency reliability, convergent validity, and construct validity were found among Japanese men and women. However, the current study did not investigate cross-cultural validity, interpretability, or responsiveness.

### Validity

A series of confirmatory factor analyses using the multi-group method revealed that the four-factor model (Model 4) fitted the data better than the one-factor model (Model 3). In addition, as some intervention studies have been implemented using the FSSB with four factors (Hammer et al., 2011, 2016; McHale et al., 2016), FSSB was treated as a four-factor model, in line with the original study of FSSB (Hammer et al., 2009). This feature of the present study facilitates comparability between the current results and previous research findings.

Family demands and work–life conflict differ according to social and cultural context (Annor and Burchell, 2018). In Asian cultures, females and males typically play substantially different family roles, and exhibit differences in family- and work-related values. In the Global Gender Gap Index in 2018, Asian countries typically ranked lower than western countries (World Economic Forum, 2018). Japanese women tend to bear the majority of the responsibility for child care and housekeeping, even when employed outside the home. Female workers spend an average of 208 min per day on child care and housekeeping, compared with 44 min per day for men (Statistics Bureau Japan Ministry of Internal affairs and Communications, 2017). Contrary to expectations, the four-factor structure was invariant between men and women in the current study; that is, the factor loading of the items on the underlying factors did not differ systematically between men and women. Future research should examine gender differences in the impact of FSSB in an international context.

To provide evidence of construct validity, FSSB-J scores were used as predictors of six important aspects of work–family constructs (i.e., work–family positive spillover, family–work positive spillover, work engagement, intention to leave, job satisfaction, and psychological distress). FSSB was significantly and negatively correlated with intention to leave and psychological distress, and positively correlated with procedural justice, interactional justice, supervisor support, coworker support, WFPS, FWPS, work engagement, and job satisfaction. These results are consistent with previous studies (Hammer et al., 2009, 2011; Crain et al., 2014). Thus, FSSB-J scores have significant construct and convergent validity with respect to all important variables.

### Reliability

The internal consistency values of each of the four dimensions were excellent (0.90 < α < 0.94). These values are comparable to or higher than those reported in the original study of the FSSB (0.74 < α < 0.90) (Hammer et al., 2009). Moreover, test–retest reliability was high and the latent factor structure of FSSB was relatively stable over time. Thus, the Japanese version of FSSB appears to have a level of reliability that is comparable to the original version.

### Limitations and Future Directions

The present study involved several limitations. First, all measures used in this study were self-reported, which could be a source of bias. Common method variance may have affected the results, suggesting that the true associations between variables may be weaker than those observed in this study. Future studies could use objective measures (e.g., objective company records and other records that capture work–life balance) to further investigate this construct. Second, this study used a convenience sample of people registered with an online survey company, and so may not be a representative sample of the Japanese working population. This could be a concern, as the reliability and validity of scales have been reported to depend on the characteristics of the sample (Mokkink et al., 2018). Third, this study included only married workers and did not take into account the possibility that unmarried individuals may also have family needs and work–family conflicts. The generalizability of the results is unclear, because FSSB is highly dependent on the context of the organization. Finally, the sample was predominantly male (∼70% of the sample in both surveys), potentially limiting the generalizability of the current findings across genders.

## Conclusion

The current study suggests that the FSSB-J adequately measures FSSB, and can be used effectively in the Japanese context. The introduction of this questionnaire in Japan should stimulate further research on work–life balance in Japan and also international research collaborations on work–life balance.

## Data Availability Statement

The datasets generated for this study are available on request to the corresponding author.

## Ethics Statement

The studies involving human participants were reviewed and approved by the Ethics Committee of the Graduate School of Medicine and Faculty of Medicine at The University of Tokyo [11666-(1)]. Written informed consent for participation was not required for this study in accordance with the national legislation and the institutional requirements.

## Author Contributions

HE contributed to the conception and design of the study, performed the statistical analysis, and wrote the first draft of the manuscript with support from YK, HK, MS, MT, and AS. MS and MT organized the database. YK contributed to translate into Japanese. HK contributed to the back translation. AS was involved in planning and supervised the work. All authors contributed to manuscript revision, and read and approved the submitted version of the manuscript.

## Conflict of Interest

The authors declare that the research was conducted in the absence of any commercial or financial relationships that could be construed as a potential conflict of interest.
